# Supplementary material for: Health Risk Assessment of Exposure to 15 Essential and Toxic Elements in Spanish Women of Reproductive Age: A Case Study
Source: Int J Environ Res Public Health. 2021 Dec 9;18(24):13012. doi: 10.3390/ijerph182413012 (PMC8701213; doi:10.3390/ijerph182413012)
Supplement: Supplementary file 1 [file ijerph-18-13012-s001.zip › ijerph-1460915-supplementary.pdf]

# Supplementary Information

# Exposure and Risk Assessment to 15 toxic and essential elements in Spanish women of reproductive age: a case study.

**Carmen Sáez[1,2], Alfredo Sánchez[1], Vicent Yusà[3,4,5], Pablo Dualde[3], Sandra F. Fernández[3,5], Antonio López[3], Francisca Corpas-Burgos[3] , Miguel Ángel Aguirre[2] and Clara Coscollà[3]***

[1] Public Health Laboratory of Alicante, 6 Plaza de España, 03010 Alicante, Spain
[2] Department of Analytical Chemistry, Nutrition and Food Science, University Institute of Materials, University of Alicante, P.O. Box 99, 03080 Alicante, Spain
[3] Foundation for the Promotion of Health and Biomedical Research in the Valencian Region, FISABIO-Public Health, 21, Avenida Catalunya, 46020, Valencia, Spain.
[4] Public Health Laboratory of Valencia, 21, Avenida Catalunya, 46020 Valencia, Spain.
[5] Analytical Chemistry Department, University of Valencia, Edifici Jeroni Muñoz, Dr. Moliner 50, 46100 Burjassot, Spain.
* Correspondence: coscolla_cla@gva.es; Tel.: +34 961926333; Fax: +34 961925704

**Table S1.** Analysis of metals in urine by ICP-MS. Determination mode and LoQ.

| Analyte | Mass (amu) | Mode | Internal Standard | LoQ (µg/L) |
|---|---|---|---|---|
| Aluminium | $^{27}$Al | Standard | $^{45}$Sc | 5.0 |
| Barium | $^{137}$Ba | Standard | $^{89}$Y | 0.021 |
| Beryllium | $^{9}$Be | Standard | $^{45}$Sc | 0.012 |
| Cobalt | $^{59}$Co | DRC | $^{72}$Ge | 0.008 |
| Caesium | $^{133}$Cs | Standard | $^{89}$Y | 0.005 |
| Copper | $^{65}$Cu | DRC | $^{72}$Ge | 0.055 |
| Manganese | $^{55}$Mn | DRC | $^{72}$Ge | 0.012 |
| Nickel | $^{60}$Ni | DRC | $^{72}$Ge | 0.053 |
| Lead | $^{206,207,208}$Pb | Standard | $^{209}$Bi | 0.014 |
| Platinum | $^{195}$Pt | Standard | $^{193}$Ir | 0.004 |
| Antimony | $^{121}$Sb | Standard | $^{72}$Ge | 0.009 |
| Thorium | $^{232}$Th | Standard | $^{209}$Bi | 0.013 |
| Uranium | $^{238}$U | Standard | $^{193}$Ir | 0.005 |
| Vanadium | $^{67}$VO | DRC | $^{109}$NbO | 0.064 |
| Zinc | $^{66}$Zn | DRC | $^{72}$Ge | 0.228 |

**Table S2.** ICP-MS instrumental operating conditions.

| Component/Parameter | Type/Value/Mode |
|---|---|
| Nebulizer | PFA standard |
| Spray Chamber | Glass cyclonic, baffled and cooled |
| Sampler and Skimmer Cones | Platinum |
| Plasma Gas Flow | 15 L/min |
| Auxiliary Gas Flow | 1.2 L/min |
| Nebulizer Gas Flow | 0.90-0.95 L/min |
| Sample Uptake Rate | 1.00 mL/min |
| RF Power | 1200 W |
| Sweeps | 20 |
| Points per peak | 1 |
| Replicates per Sample | 4 |
| Dwell time | 100 ms (50 ms for IS) |
| Integration Time | 2000 ms (1000 ms for IS) |
| Mode of Operation | Standard and DRC (using $CH_4$ and $O_2$) |

**Table S3.** Determined limits of detection (LoD) and limits of quantification (LoQ).

| Analyte | LoD (µg/L) | LoQ (µg/L) |
|---|---|---|
| Al | 1.6 | 5.0 |
| Ba | 0.006 | 0.02 |
| Be | 0.004 | 0.012 |
| Co | 0.002 | 0.008 |
| Cs | 0.002 | 0.005 |
| Cu | 0.016 | 0.06 |
| Mn | 0.004 | 0.012 |
| Ni | 0.016 | 0.05 |
| Pb | 0.004 | 0.014 |
| Pt | 0.001 | 0.004 |
| Sb | 0.003 | 0.009 |
| Th | 0.004 | 0.013 |
| U | 0.002 | 0.005 |
| V | 0.019 | 0.06 |
| Zn | 0.07 | 0.2 |

**Table S4.** Results for the Clinchek - Control / Urine control lyophilised / RECIPE® Level I and II.

| Analyte | Level I | | Level II | |
|---|---|---|---|---|
| | Obtained value (μg/L) | Certified range (μg/L) | Obtained value (μg/L) | Certified range (μg/L) |
| Al | 78.2 | 76.2 – 114.2 | 133 | 118 – 178 |
| Ba | 2.07 | (1.46 - 2.18)* | 11.7 | (8.6 - 13.0)** |
| Be | 1.01 | (0.84 - 1.26)* | 9.8 | (8.0 - 12.0)** |
| Co | 2.11 | 1.80 - 2.70 | 34.0 | 28.5 - 42.7 |
| Cs | 2.26 | (1.70 - 2.54)* | 11.5 | (8.9 - 13.3)** |
| Cu | 58.2 | 46.9 - 70.3 | 108.3 | 88.8 – 133 |
| Mn | 3.66 | 3.34 - 5.02 | 19.1 | 15.8 - 23.6 |
| Ni | 6.42 | 4.98 - 7.46 | 41.8 | 35.8 - 53.6 |
| Pb | 20.2 | 19.2 - 28.8 | 45.9 | 51.4 - 77.2 |
| Pt | 1.08 | (0.84 - 1.26)* | 10.2 | (8.0 - 12.0)** |
| Sb | 11.4 | 9.6 - 14.4 | 44.3 | 38.2 - 57.4 |
| Th | 1.22 | (0.86 - 1.30)* | 10.2 | (8.0 - 12.0)** |
| U | 1.05 | (0.80 - 1.20)* | 9.9 | (8.0 - 12.0)** |
| V | 21.3 | 15.7 - 23.5 | 53.6 | 38.4 - 57.6 |
| Zn | 188 | 161 - 269 | 465 | 429 - 643 |

()* denotes sample spiked with 1 μg/L and ()**denotes sample spiked with 10 μg/L.
